# Involvement of the Estrogen and Progesterone Axis in Cancer Stemness: Elucidating Molecular Mechanisms and Clinical Significance

**DOI:** 10.3389/fonc.2020.01657

**Published:** 2020-09-04

**Authors:** Bi Chen, Peng Ye, Yeh Chen, Tong Liu, Jong-Ho Cha, Xiuwen Yan, Wen-Hao Yang

**Affiliations:** ^1^Affiliated Cancer Hospital and Institute of Guangzhou Medical University, Guangzhou, China; ^2^Institute of New Drug Development, China Medical University, Taichung, Taiwan; ^3^Department of Breast Surgery, Harbin Medical University Cancer Hospital, Harbin, China; ^4^The Institute of Cancer Prevention and Treatment, Harbin Medical University, Harbin, China; ^5^Department of Biomedical Sciences, College of Medicine, Inha University, Incheon, South Korea; ^6^Graduate Institute of Biomedical Sciences, China Medical University, Taichung, Taiwan

**Keywords:** cancer stem cells, cancer stemness, estrogen, female hormone, progesterone

## Abstract

Estrogen and progesterone regulate the growth and development of human tissues, including the reproductive system and breasts, through estrogen and progesterone receptors, respectively. These receptors are also important indicators for the clinical prognosis of breast cancer and various reproductive cancers. Many studies have reported that cancer stem cells (CSCs) play a key role in tumor initiation, progression, metastasis, and recurrence. Although the role of estrogen and progesterone in human organs and various cancers has been studied, the molecular mechanisms underlying the action of these hormones on CSCs remain unclear. Therefore, further elucidation of the effects of estrogen and progesterone on CSCs should provide a new direction for developing pertinent therapies. In this review, we summarize the current knowledge on the estrogen and progesterone axis involved in cancer stemness and discuss potential therapeutic strategies to inhibit CSCs by targeting relevant pathways.

## Introduction

Steroid hormones play a major role in the pathogenesis and progression of breast, ovarian, and other types of cancers. They can be simply divided into sex hormones, which include male hormones (androgen) and female hormones (estrogen and progesterone), and corticosteroids, which are grouped into glucocorticoids and mineralocorticoids. These hormones generally regulate cell function by activating nuclear steroid receptors, which can be classified into five types: estrogen receptors (ERs), progesterone receptors (PRs), androgen receptors, glucocorticoid receptors, and mineralocorticoid receptors (1). However, steroid hormones can achieve rapid regulation of cell activity through membrane receptors (2). Emerging research has shown that female hormones are involved in the proliferation, differentiation, and metastasis of cancer stem cells (CSCs), which can self-renew, possess a tumor-initiating ability, and contribute to tumor development and recurrence. Their phenotype is dynamically regulated by cell signaling transduction and the tumor microenvironment (TME) (3, 4). In the TME, tumor-associated fibroblasts (TAFs), macrophages (TAMs) and mesenchymal stem cells secrete interleukin 6 (IL-6), interleukin 8 (IL-8), and chemokine (C-X-C motif) ligand 7 (CXCL7), stimulating the self-renewal of CSCs (3). In addition, the TME comprises stromal cells and secretes various cytokines, growth factors, and proteases, including platelet-derived growth factor (PDGF), vascular endothelial growth factor (VEGF), and matrix metalloproteinases. These proteins promote tumor invasion and eventual metastasis (5, 6). Various cancers of the reproductive organs are particularly vulnerable to estrogen and progesterone through ERs and PRs, respectively. In the classical model for steroid hormone signaling, hormones enter the cells through binding to the compatible receptors on the plasma membrane, leading to the internalization. These complexes then bind directly to DNA response elements, such as estrogen response elements, and regulate the nuclear transcription of target genes, thereby changing the biological response of CSCs (7–9). Estrogen and progesterone also participate in the regulation of CSC populations through a paracrine manner (10, 11). Moreover, microRNAs (miRNAs) are involved in the regulation of the sex hormone axis in CSCs. They are small non-coding RNAs that can silence their cognate target genes by degrading mRNA molecules or inhibiting their translation (12). MiRNAs have been reported to contribute to the regulation of cancer stemness and metastasis, suggesting that they may function as oncogenes or tumor suppressors (13, 14). Both estrogen and progesterone can regulate self-renewal of CSCs by miRNA expression (15–18).

Hormone-related cancers such as breast cancer depend on estrogen signaling and therefore respond to endocrine therapies that block estrogen signaling. Endocrine therapy is a key treatment for hormone-related cancers and has proven clinical benefits (19, 20). However, hormone-related tumors often show endocrine resistance. Numerous mechanisms, including receptor mutations (21, 22) and crosstalk in other signaling pathways (23), have been proposed to explain the emergence of endocrine resistance. Increasing evidence suggests that CSCs play a critical role in endocrine therapy resistance (24–27). During the formation of CSC-like cells, cancer cells undergo cell reprogramming, which resets the differentiated cells to a pluripotent state through nuclear transfer, cell fusion, and overexpression of transcription factors, such as octamer-binding transcription factor 4 (Oct-4), sex-determining region Y-Box 2 (SOX2), Kruppel-like factor 4 (KLF4), and c-MYC (or OSKM) (28). Also known as stemness markers, these transcription factors are associated with endocrine therapy resistance (29, 30). The mechanism of endocrine resistance in CSCs also includes the upregulation of self-renewal signaling pathways such as Wnt and Hedgehog (31–33). Progesterone and estrogen are the most essential hormones that regulate the stem cells of the human reproductive system (34–36). They can induce CSC proliferation and increase the risk of reproductive cancers (17, 37, 38). Therefore, both estrogen and progesterone appear to be key regulators that control the number and function of CSCs. It is thus pivotal to explore the relationship between female hormones and CSCs to develop more cancer therapies that target CSCs.

## Characteristics of Stemness in Cancer Progression

CSCs, a subpopulation of cancer cells that can self-renew, exist in most cancer types and promote tumorigenesis, tumor drug resistance, metastasis, and relapse. They are also known as tumor-initiating or sphere-forming cells, and they can be isolated from most types of human cancers, including breast, brain, liver, lung, stomach, colon, prostate, pancreas, and head and neck cancers (39). CSCs usually have some characteristics in common with normal stem cells, including relative quiescence, an active DNA repair system, aggressiveness, and drug resistance (40, 41). Tumor resistance to radiotherapy and chemotherapy and recurrence can be attributed to the presence of CSCs (42). The reason why CSCs are inherently resistant to chemotherapy and radiotherapy is mainly because of quiescence, during which CSCs exhibit a slow growth rate, with the cell population maintained in the G0 phase (43). In addition, CSCs have the plasticity to change from the state of quiescence to that of continuous differentiation and proliferation in response to conventional chemotherapy and radiotherapy, leading to tumor recurrence (44). Some studies have also reported that CK5^+^ breast cancer cells possess CSC-like properties and that progesterone treatment can induce the conversion of ER^+^/PR^+^/CK5^−^ cells to ER^−^/PR^−^/CK5^+^ cells, which represent a relatively quiescent state, leading to resistance to endocrine therapy and chemotherapy (45–47). These findings imply that female hormones regulate the fate of CSCs.

Because CSCs display plasticity and are a small population of cells within a tumor, it is difficult to accurately identify and eradicate them. At present, the expression of cell surface markers is often used to identify CSCs in preclinical cell models (48). In patients with colorectal cancer, Lgr5 is an important biomarker of colorectal CSCs, and detecting Lgr5^+^ CSCs is a crucial indicator to predict tumor recurrence (49). ATP-binding cassette subfamily G member 2 (ABCG2) has been found to play an integral role in the molecular mechanisms underlying multidrug resistance in CSCs (50). B-cell specific Moloney murine leukemia virus integration site 1 (BMI-1) is a stemness-related biomarker that can maintain self-renewal of CSCs (39, 51). CXC chemokine receptor 4 (CXCR4) is an another stemness-related biomarker associated with tumor growth, invasion, metastasis, and relapse (39, 52). Moreover, cell surface markers such as CD24, CD26, CD44, CD90, CD133, CD166, CD177, aldehyde dehydrogenase 1 (ALDH1), and epithelial cell adhesion molecule (EPCAM) have been identified as biomarkers of CSCs in various types of cancers (39, 53–55). Table 1 presents a summary of the currently known CSC biomarkers and their characteristics. CSCs can, however, also be found in single CSC marker–negative cell populations, which means that any single CSC marker does not cover all CSC populations. Therefore, multiple CSC markers are needed to encompass most CSC subsets. For example, CD44^+^/CD24^−^/ALDH1^+^ breast cancer cells represent the most enriched set of breast cancer stem cells (BCSCs). This method of combining multiple CSC markers to define CSC populations is thus highly predictive of tumor malignancy (56).

**Table 1:** CSC biomarkers and their related characteristics.

**CSC biomarkers**	**Characteristics**	**Cancer tissues**	**References**
CD133	Cell growth and differentiation, inhibits apoptosis	Breast, lung, prostate, ovary, liver, colon, and pancreas	(56, 57)
CD44	Cell division, migration, adhesion, and signaling	Breast, lung, prostate, ovary, stomach, colon, liver, and head and neck	(51, 58)
CD24	Cell migration and proliferation	Breast, ovary, stomach, and pancreas	(51, 59, 60)
CD49f	Cell differentiation, proliferation, and metastasis	Breast	(61, 62)
ABCG2	Intracellular transport	Breast, lung, ovary, pancreas, and liver	(49)
CD90	Cell differentiation	Breast, brain, liver, and lung	(38, 51)
EPCAM	Cell migration, proliferation, adhesion, and signal	Breast, lung, ovary, colon, and pancreas	(38, 63)
	transduction		
ALDH	Cell migration, invasion, and metastasis	Breast, lung, prostate, bladder, stomach, colon, and head and neck	(38, 64)
Lgr5	G protein coupled receptors, promote cell proliferation	Colon, stomach, and head and neck	(48)
CD177	Tyrosine kinase receptor	Leukemia, lung	(54)
CXCR4	Chemokine receptor	Breast, glioma, and pancreas	(51)
BMI-1	Maintain CSC self-renewal	Breast, leukemia, pancreas, prostate, head and neck, and lung cancer	(38, 39)

*CD133, Cluster of differentiation 133; CD44, Cluster of differentiation 44; CD24, Cluster of differentiation 24; CD49f, Cluster of differentiation 49f; ABCG2, ATP-binding cassette sub-family G member 2; CD90, Cluster of differentiation 90; EPCAM, epithelial cell adhesion molecule; ALDH, aldehyde dehydrogenase; Lgr5, Leucine-rich repeat-containing G-protein coupled receptor 5; CD177, Cluster of differentiation 177; CXCR4, CXC chemokine receptor 4; BMI-1, B-cell specific moloney murine virus integration site 1*.

Because CSCs promote tumor recurrence and progression, it is particularly important to eradicate them. At present, many drugs against CSCs have been developed that target the mechanisms regulating CSCs. For example, drugs targeting CSC-associated surface markers include anti-EPCAM, anti-CD16, and anti-CD47. Clinical trials have also investigated other anti-CSC drugs that target developmental pathways, upregulated apoptotic pathways, the TME, and upregulated drug efflux pumps (44). However, because these cells display plasticity, no specific surface markers have been identified as yet; furthermore, considering the complexity of the regulatory pathways involved in their regulation, it is challenging to completely eradicate CSCs. Therefore, we must obtain a better understanding of the regulatory mechanisms of CSCs to develop more effective therapies for eliminating them. Herein we focus on discussing the effects of estrogen and progesterone on CSCs.

## Regulatory Networks of the Estrogen Axis in CSCs

Estrogen exerts its biological function by binding to ERs, which are generally composed of membrane ERs (mostly G protein–coupled receptors) and nuclear ERs (ERα and ERβ) (65, 66). Estrogen primarily regulates CSCs through these receptors.

**Figure 1:**
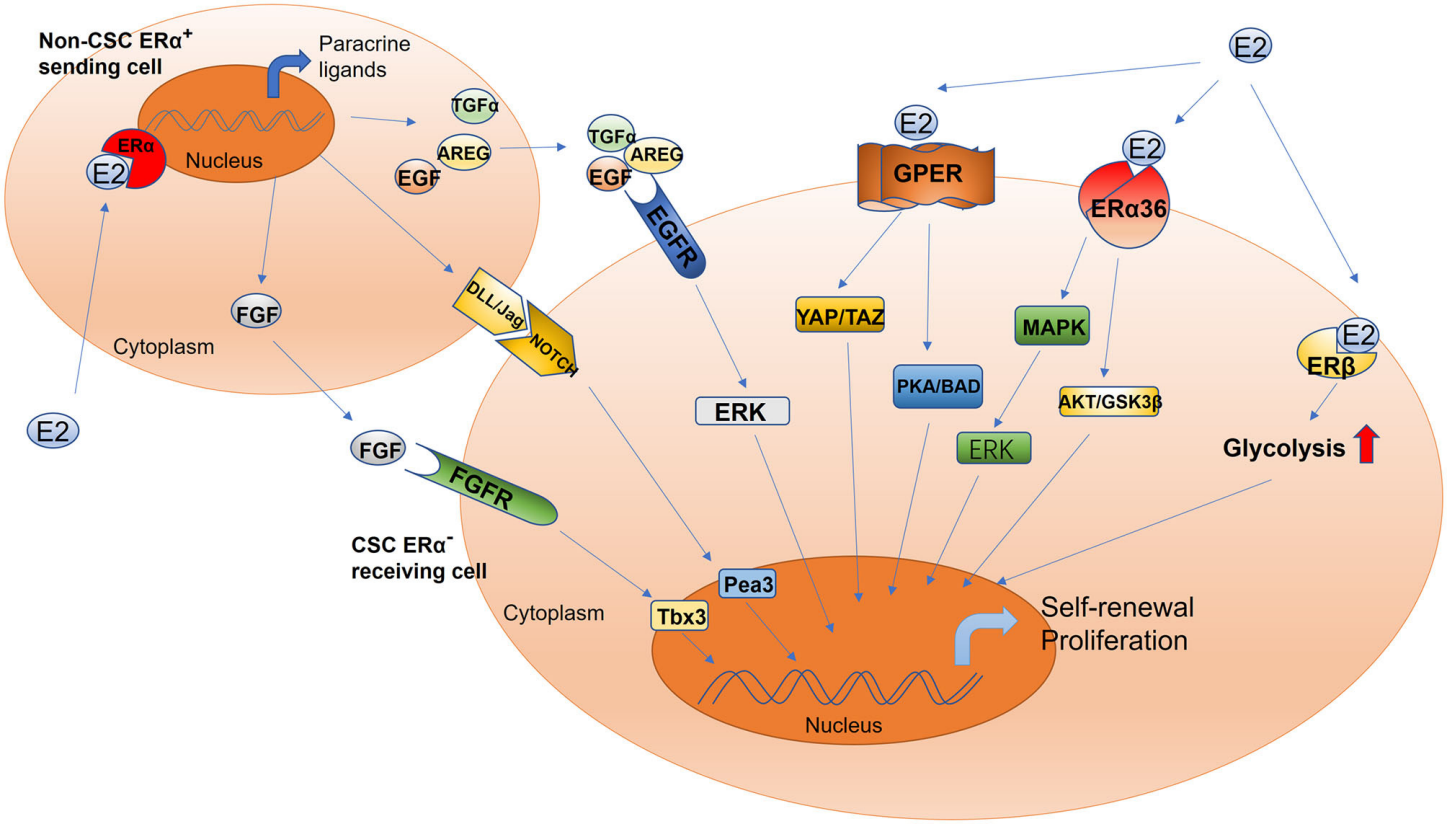
Schematic of estrogen-mediated paracrine signaling and major pathways for the activation of different estrogen receptor isoforms in CSCs. The sending cell (non-CSC) responds to estradiol and initiates the production of the fibroblast growth factor receptor, epidermal growth factor receptor, and Notch ligands. The signal cascade is initiated within the receiving cell (CSC), including but not limited to ERK, Tbx3, and Pea3 signaling. This mechanism drives increased CSC activity. Estrogen promotes the proliferation and self-renewal of CSCs through different estrogen receptor isoforms, including GPER, ERα36, and ERβ. GPER, G-protein coupled receptor; ERα36, estrogen receptor alpha 36; ERβ, estrogen receptor beta; YAP, Yes-associated protein; TAZ, tafazzin; PKA, protein kinase A; BAD, Bcl-2 antagonist of cell death; AKT, also known as protein kinase B; GSK-3β, glycogen synthase kinase-3 beta; ERK, extracellular signal-regulated kinase; MAPK, mitogen-activated protein kinase; Tbx3, T-box transcription factor; Pea3, polyomavirus enhancer activator 3; AREG, amphiregulin; TGFα, transforming growth factor alpha; FGF, fibroblast growth factor; EGF, epidermal growth factor; DLL/JAG, Notch ligands Jagged (Jag) and Delta-like (Dll).

The conventional model of estrogen signaling is direct genomic signaling; in this process, estrogen binds to ERα or ERβ to promote DNA transcription in the nucleus (66). ERα and ERβ share common structural features that are characterized by several functional domains, and they maintain receptor-specific signal transduction through exclusive elements (65, 67, 68). Estradiol (E2) is a steroidal estrogen with two subtypes, 17α-estradiol (17α-E2), and 17β-estradiol (17β-E2). 17α-E2 is a naturally occurring enantiomer of 17β-E2 and possesses low activity to activate ERα and ERβ, the classical estrogen receptors. The downstream signaling and physiological functions of endogenous 17α-E2 are unclear (69, 70). Rather than 17α-E2, 17β-E2 is generally considered to be the physiological form of estrogen; it typically activates estrogen receptors due to its high affinity with ERα and ERβ (69). Furthermore, because 17β-E2 has a higher binding affinity for ERα than for ERβ, estrogen mainly functions in concert with ERα to perform its biological functions (71, 72). For example, estrogen induces the binding of ERα to the promoter region of piwi-like RNA-mediated gene silencing 1 (PIWIL1), a critical gene for stem cell self-renewal, leading to the overexpression of PIWIL1 in endometrial cancer cells and stimulating cancer cell proliferation (73, 74). However, most CSCs are ER^−^; accordingly, the estrogen signal is mediated through paracrine signaling from non-CSCs (expressing ER or PR) to CSCs (10, 75, 76). The fibroblast growth factor (FGF)/Tbx3 signaling, epidermal growth factor (EGF), and Notch signaling pathways operate downstream of estrogen in the regulation of ER^−^ CSCs (77, 78) (Figure 1). In addition, estrogen can reduce the proliferation and self-renewal capacity of CSCs by downregulating the embryonic stem cell genes *NANOG, OCT4*, and *SOX2* (79). This explains why patients with an ER^+^ tumor tend to have better prognoses than other patients (80, 81). Contradictory results reported by these studies can be attributed to differences in experimental designs. Moreover, a few studies have reported that estrogen itself does not change the stem characteristics of stem or progenitor cells (45, 46, 82). Therefore, further studies are warranted to validate the effects of estrogen on CSCs.

Although estrogen can bind to nuclear ERs to regulate CSCs, this nuclear, transcriptional action cannot account for all the biological functions of ERs (66). Although most CSCs are ER^−^ (10, 75), estradiol can nevertheless increase the number of CSCs, possibly *via* the non-genomic signaling pathway, which is a regulatory mechanism that does not involve direct binding to DNA and only occurs in the plasma membrane and cytoplasm (66, 83, 84). The non-genomic estrogen signals that regulate CSCs are mainly mediated by cell membrane–associated ERs, including G protein–coupled ER (GPER/GPR30) (85–87), ERα variant ERα36 (88–90), and ERβ (91) (Figure 1).

The estrogen membrane receptor GPER/GPR30 can be activated by estrogen and mediates rapid non-genomic signaling. GPER/GPR30 is a transmembrane receptor expressed by both ER^+^ and ER^−^ breast cancer cells and plays a role in breast cancer development (92). Hu et al. demonstrated that estrogen can activate tafazzin (TAZ) through GPR30 to regulate the Hippo pathway and promote breast cancer cell proliferation and migration as well as tumor growth (86). TAZ, a GPER-induced transcription coactivator, is also associated with the self-renewal and tumor-initiating capacities of CSCs (93). TAZ is highly expressed in BCSCs; the downregulation of TAZ expression in these cells can significantly reduce their sphere-forming ability and chemotherapy resistance (94, 95). Further, estrogen can reportedly increase the number of CSCs by activating TAZ, thereby promoting chemotherapy resistance. Huang et al. proved that GPER activation by estrogen suppressed triple-negative breast cancer (TNBC) MDA-MB-231 cell proliferation, migration, invasion, and angiogenesis as well as the process of epithelial–mesenchymal transition by regulating the miR-199a-3p/CD151 axis and inactivating the Hippo signaling pathway; they also found that miR-199a-3p overexpression distinctly inhibited the Hippo pathway by downregulating the expression of Yes-associated protein 1 (YAP1) in MDA-MB-231 cells (85). YAP1 is a major downstream effector of the Hippo pathway; it is a recognized oncogene and plays a key role in regulating stem cell self-renewal and differentiation (96–100). Therefore, estrogen's ability to activate GPER to regulate the CD151/miR-199a-3p biological axis and inhibit the Hippo pathway may be related to its role in regulating CSCs. However, further studies are needed to validate the accuracy of this hypothesis. In addition, Chan et al. demonstrated that estrogen activates GPER-mediated protein kinase A (PKA)/Bcl-2 antagonist of cell death (BAD) signaling, and this also plays a crucial role in maintaining the stemness of BCSCs. GPER activation by estrogen reportedly induces PKA and BAD–Ser118 phosphorylation to promote the proliferation of BCSCs (87). In general, estrogen can positively regulate CSCs through GPER/GPR30; although the underlying mechanism is not particularly well-understood, it can still provide clues to facilitate the development of targeted drugs.

The most commonly found isoform of ERα is the 66-kDa protein ERα66; other forms include ERα36, a variant of ERα with a molecular weight of 36 kDa, which is a plasma membrane–based receptor in some human tissues and breast cancer cell lines with or without ERα66 (90, 100, 101). Despite ERα36 lacking both AF-1 and AF-2 transactivation domains of the full-length ERα66 and transcriptional activity, it retains DNA binding, dimerization, and ligand-binding domains (102, 103). In the absence of full-length ERs, estrogen can mediate rapid non-genomic estrogen signals to regulate CSCs through ERα36 (90). ERα36-mediated rapid estrogen signaling *via* the AKT/GSK3β pathway is known to positively regulate ER^+^ breast cancer stem/progenitor cells (88). ERα36 can also enhance the self-renewal capacity of CSCs to promote tamoxifen resistance; ERα36 downregulation can significantly reduce the number of CSCs and block an increase in their population and the formation of tumorspheres, thus overcoming tamoxifen resistance (88, 104, 105). Increased expression of ERα36 is one of the underlying mechanisms of tamoxifen resistance, and this indicates that ERα36 can serve as a therapeutic target for inhibiting this resistance (106, 107). In addition, knocking out the expression of ERα36 in the HER2^+^ breast cancer cell line SKBR3 has been reported to reduce HER2 expression, and the number of ALDH^High^ cells also decreases (89). In summary, estrogen-activated ERα36 can positively regulate CSCs, and ERα36 downregulation can suppress cancer stemness (108). Thus, ERα36 can be used to target CSCs (109, 110).

ERβ is mainly expressed in the nucleus, but it can also be found in the cytoplasm and plasma membrane, where it can mediate non-genomic estrogen signals (111). In comparison with ERα, the expression level of ERβ evidently decreases with the progression of cancer. Even so, ERβ participates in regulating CSCs, and thus, it is gradually attracting research attention (110). Through single-cell analysis, human BCSCs sorted by fluorescence-activated cell sorting have been compared with total tumor cells, and the expression of the ERβ gene was found to be significantly upregulated (112). With regard to the potential of ERβ as a stemness marker, Ma et al. found that the expression of ERβ was closely related to that of the CSC markers CD44 and ALDH1 in the absence of ERα and is essential for the growth of mammospheres (113). Moreover, ERβ is reportedly responsible for the upregulation of glycolysis. The maintenance of the phenotype of BCSCs depends on ERβ-mediated glycolysis. Thus, ERβ can be considered a stemness marker in CSCs (113). Human prostate CSCs and papillary thyroid CSCs highly express ERβ (114, 115). ERβ overexpression in CSCs can promote cancer stemness through estrogen signaling (91, 115). Thus, estrogen can directly regulate CSCs by activating ERβ. However, contradictory results have been reported too: some studies have suggested that ERβ is responsible for repressing proliferation and inducing apoptosis (116, 117) and that upregulated ERβ levels are positively correlated with better disease-free survival (118). Moreover, many studies have reported that activated ERβ inhibits cancer stemness and induces apoptosis of CSCs in mouse as well as human tumor models (88, 89). Based on the inhibitory effects of ERβ on CSCs and considering that the current method of managing prostate diseases is to prevent cell regeneration, targeting ERβ appears promising (119, 120). At present, the known ERβ signaling mechanism in breast and other types of cancers is not as clear as the ERα signaling mechanism; therefore, the regulation of CSCs by ERβ signaling demands further explorations.

Estrogen can also affect cancer stemness by regulating the expression of miRNA. For example, estrogen has been reported to increase the expression of miR-21 and reduce self-renewal of cancer cells with stem cell–like properties by inhibiting the translation of the stem cell genes *Oct-4, c-Myc, Nanog*, and *Sox2* (15). In addition, stimulating ERα^+^ breast cancer cells with estrogen reportedly promotes tumor-initiating cell renewal *via* the suppression of miR-140 expression (16). Moreover, ovarian cancer may be caused by ovarian cancer-initiating cells characterized by surface antigen CD44 and the ovarian CSC maker c-KIT (CD117). Estrogen-induced expression of the transcription factor E2F6 by inhibiting miRNA-193a activity upregulates c-KIT to promote ovarian tumorigenesis (121, 122). Thus, estrogen can regulate the formation and differentiation of CSCs by affecting the expression level of miRNA.

Altogether, the signal transduction of estrogen in CSCs is complex, and accordingly, more studies need to be conducted to completely elucidate the effects of estrogen on CSCs. Whether ERs can be used as a target to steadily regulate CSCs also demands further investigations.

## Effects of Anti-estrogen Drugs on CSCs

The effects of estrogen on CSCs are not completely clear. The regulation of CSCs by estrogen requires ERs. At present, anti-estrogen drugs that target and regulate ER activity are mainly divided into three categories: selective ER modulators (SERMs), selective ER downregulators (SERDs), and aromatase inhibitors (123). Using these to treat patients with ER^+^ tumors has undoubtedly led to great success, but endocrine resistance often occurs post treatment. As most CSCs are ER^−^, they are not the targets of anti-estrogen therapy. With these treatments, not only are CSCs uninhibited but also significantly enriched. Letrozole, an aromatase inhibitor, can stimulate an increase in the number of CD44^+^/CD24^−^ breast cancer cells and the formation of mammospheres after treatment (124). In addition, anti-estrogen treatment (tamoxifen) can increase the number of CSCs and promote formation of tumorspheres. Further, it has been reported that the expression of the embryonic stem cell marker SOX2 and subsequent activation of the WNT signaling pathway play a key role in inducing drug resistance after tamoxifen treatment (125). Moreover, treatment with the anti-estrogen drugs tamoxifen or fulvestrant decreases cell proliferation but increases the BCSC population through JAG1–NOTCH4 receptor activation (10). Altogether, these findings confirm that inhibiting estrogen signaling in cancer cells may promote the stemness of CSCs, causing resistance to endocrine therapies. Treating patients with cancer using anti-estrogen drugs alone is not ideal. Identifying additional mechanisms responsible for resistance to endocrine therapy and combining anti-estrogen therapy with blockade of these mechanisms are bound to increase treatment efficacy (78, 126).

## Blockade of Cancer Stemness by Targeting the Estrogen Axis

Anti-estrogen drugs do not effectively inhibit CSCs according to current research and thus have limited clinical application. These drugs often lead to drug resistance and tumor recurrence in patients with cancer. Therefore, new therapeutic strategies and therapies that can effectively and specifically block estrogen-induced cancer stemness are urgently required. In recent years, other drugs or genes have also been studied for their potential to block estrogen-induced cancer stemness. Oct-4 is a key transcription factor associated with the pluripotent and self-renewal characteristics of embryonic stem cells, germ cells, and adult human stem cells (127, 128). The ablation of Oct-4 expression in MCF-7 breast cancer cells causes apoptosis of CSCs and inhibits tumor growth (129). Metformin, an antidiabetic drug, has recently been reported to reduce tumor risk in some cancers associated with diabetes, including breast cancer (130, 131). Metformin can also inhibit the expression of Oct-4 in estrogen-induced CD44^+^/CD24^−/low^ MCF-7 cells and significantly reduce the size and number of their mammospheres (132). Melatonin and tocopherols have also been shown to inhibit cancer stemness. These three drugs can inhibit the binding of ER-estrogen complexes to Oct-4 promoter regions to reduce Oct-4 expression, thereby inhibiting the self-renewal of CSCs (132–134). In addition, the *let-7* miRNA family is involved in carcinogenesis and tumor progression by inducing CSC differentiation. A study showed that *let-7c*, a member of the *let-7* family, inhibits estrogen-induced Wnt signaling by reducing ERα expression, then downregulates the self-renewal ability of CSCs (14). ABCG2 is used as a surface marker to isolate CSCs from cancer cells (135). Estrogen promotes cell proliferation by upregulating ABCG2, which can be suppressed by reserpine, in endometrial cancer cells (136). Although these drugs have been reported to block estrogen-induced cancer stemness, they have not been tested *in vivo*, and the studies only used a single cell line. Palbociclib, a cyclin-dependent kinase 4/6 (CDK4/6) inhibitor, has been used to treat patients with ER^+^ and HER2^−^ advanced breast cancer. Palbociclib can inhibit the expression of cyclin-dependent kinase 4 (CDK4) and reduce the proportion of estrogen-induced CSCs in ER^+^ and HER2^−^ breast cancer cell lines (137). Furthermore, the importance of phosphoinositide 3-kinase (PI3K)/protein kinase B (AKT)/mammalian target of rapamycin (mTOR) signaling for maintaining the CSC phenotype in renal cell cancer (138), prostate cancer (139), and lung cancer (140) has been confirmed. In breast cancer, the increase of CSC populations from tamoxifen treatment can be prevented with mTOR inhibitors (141). Taken together, these anti-CSC drugs are promising, but further research is required before they can be clinically used. Their underlying mechanisms should be thoroughly explored in the future, and their effectiveness and safety carefully evaluated in well-designed clinical trials.

## Regulatory Networks of the Progesterone Axis in CSCs

In the classical progesterone signaling pathway, progesterone exerts its biological functions mainly by binding to nuclear PRs. In the regulatory pathways of CSC activity, progesterone plays a role by binding to not only nuclear PRs but also cell membrane PRs (mPRs) (142). Moreover, different isoforms and post-transcriptional modification of PRs are related to CSC activity (143). However, most CSCs are PR^−^; progesterone regulates CSC activity through paracrine actions between PR^+^ and PR^−^ cells (17). Some studies have reported that progesterone can also regulate the miRNA expression involved in CSC proliferation and formation (18, 144). In addition, the complex relationship between progesterone and other hormones, such as prolactin and growth hormones (GHs), also affects CSC activity (47, 145). Considering these preclinical mechanisms for regulating CSCs, some treatment strategies can be designed to block cancer stemness by targeting the progesterone signal.

The PR^+^ phenotype of cancer cells usually indicates a good response to endocrine therapy and better prognosis in clinical tumors. However, in the case of advanced breast cancer, PRs become a critical factor that promotes the generation of CSCs and results in poor prognoses. Nuclear PRs have two isoforms: PR-A (94 kDa) and PR-B (114 kDa). These are transcribed from the same gene by two distinct promoters, resulting in different transcriptional and functional activities (146). The level and ratio of PR-A and PR-B in reproductive tissues vary based on developmental stage and hormonal status (147, 148). In normal breast cells, the isoforms are coexpressed at similar levels, but in breast cancer cells, the ratio is disrupted, with PR-A being overexpressed (35, 149). In a T47D cell model of breast cancer, PR-A was found to dominantly drive CSC expansion, and PR-B enhanced anchorage-independent proliferation. Furthermore, in comparison with PR-B^+^ tumorspheres, PR-A^+^ tumorspheres comprise more CSC populations, such as ALDH1^+^, CD44^+^/CD24^−^, and CD49f^+^/CD24^−^ cell populations, and the expression of CSC-related genes, such as FOXO1, p21, KLF4, PTGES, WNT4, and NOTCH2 is enhanced (143). In addition, high PR-A expression is more likely to cause tumor recurrence after treatment with tamoxifen (150). These results suggest that different isoforms are associated with distinct CSC populations and tumor recurrence. At present, the functional activity of progesterone is mainly assessed by measuring total PR expression, but the expression of PR isoforms is neglected. It is easy to differentiate between PR-A and PR-B using western blotting because their molecular weights markedly differ. Immunohistochemistry (IHC) can also be used to identify them in clinical biopsy specimens (151), but antibodies used for the detection of PR-A have been found to detect not only PR-A but also PR-B (152). Thus, developing new antibodies to distinguish one isoform from the other is pivotal for predicting tumor malignancy and for the clinical prognosis of patients.

The post-translation modification process of PRs can also influence the generation of CSCs. PRs undergo extensive modifications post-translation, including phosphorylation, acetylation, ubiquitination, SUMOylation, and methylation (153, 154). The phosphorylation of PR-A Ser294 is necessary for the characteristics of CSCs; the mutation of PR-A Ser294 to Ala (S294A) can evidently prevent CSC expansion and promote cancer cell proliferation (143, 155). How other post-translational modifications of PRs affect CSC activity remain unknown, and further investigations are thus warranted. Because progesterone exerts its biological function by binding to PRs, the structure and isoform of these receptors can affect progesterone-mediated functions.

**Figure 2:**
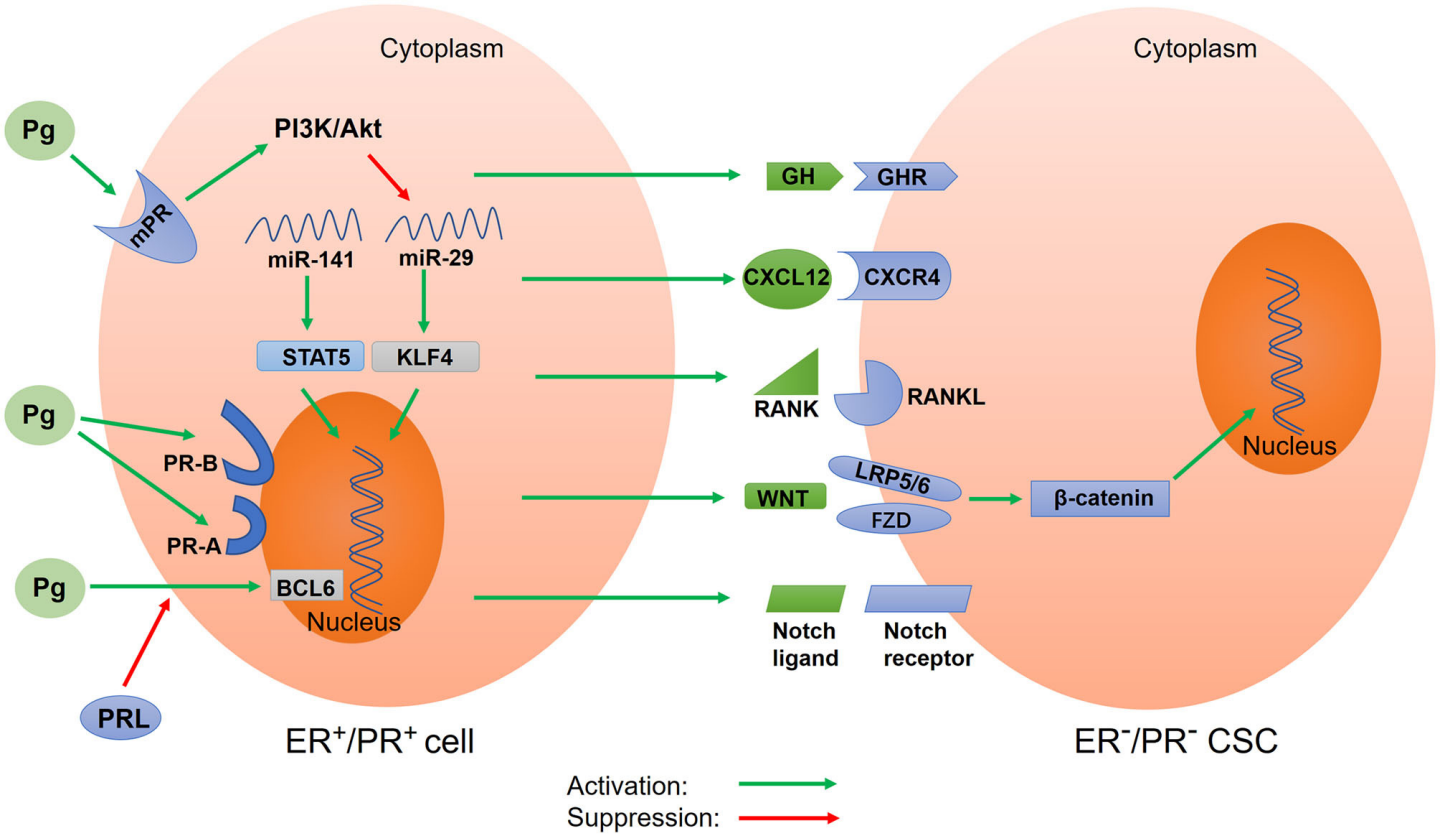
Schematic of progesterone-induced regulatory networks in CSCs. Progesterone binds to nuclear progesterone receptors (PRs) and membrane PRs, regulating the expression of target genes. Different isoforms of nuclear PR (PR-A and PR-B) alter progesterone-mediated functions in CSCs. Progesterone also regulates CSC activity *via* paracrine signals, including CXCR4/CXCL12, RANK/RANKL, WNT/β-catenin, and Notch signaling. Additionally, progesterone downregulates microRNA to increase the expression of stemness genes. Prolactin inhibits the expression of BCL6 induced by progesterone. In addition, progesterone stimulates the secretion of growth hormone (GH) to promote BCSC properties. Pg, progesterone; mPR, membrane progesterone receptor; PR-A, progesterone receptor isoform A; PR-B, progesterone receptor isoform B; PRL, prolactin; GH, growth hormone; GHR, growth hormone receptor; BCL6, B-cell lymphoma-6; CXCR4, CXC chemokine receptor type 4; CXCL12, CXC chemokine ligand 12; RANK, receptor activator of nuclear factor κB; RANKL, receptor activator of nuclear factor κB ligand; STAT5, activator of transcription 5A; KLF4, Krüppel-like factor 4.

PR-A and PR-B are key mediators in the progesterone signal. However, progesterone can also bind to mPRs to exert its biological effects through a non-classical and non-genomic mechanism (156, 157) (Figure 2). This mechanism is characterized by rapid action and does not require much time to induce the transcription and translation of target genes into proteins. The rapid non-nuclear signaling pathways activated by progesterone include the following: the extracellular signal–regulated kinase (ERK) pathway, cyclic AMP/PKA pathway, cyclic GMP/protein kinase G (PKG) pathway, Ca^++^ influx/protein kinase C (PKC) activation pathway, and phosphoinositide 3-kinase (PI3K)/Akt pathway (158). However, how these pathways affect CSC activity in tumor tissues remains unclear. In the basal-like MCF10A cells lacking nuclear PRs, progesterone activates the PI3K/Akt pathway *via* mPRs, resulting in the inactivation of *FOXO* transcriptional activity, downregulation of miRNA-29, and upregulation of *KLF4*, a transcription factor which is necessary for the maintenance of CSCs (142) (Figure 2). To summarize, the relationship between other non-nuclear signaling pathways and CSCs remains unclear and thus requires further investigations.

## Progesterone Promotes CSCs Through Paracrine Pathways

In addition to regulating CSC activity by binding to nuclear PRs and mPRs, progesterone can exert its effects through paracrine activity. Progesterone acts on PR^+^ cells to cause changes in the surrounding tumor microenvironment, thereby affecting nearby PR^−^ cells. The paracrine signals of progesterone-induced CSC expansion include the receptor activator of nuclear factor-kappa B ligand (RANKL) and WNT4 (Figure 2). The downstream component of the RANKL/RANK signal is the NF-κB pathway, which is an important pathway involved in CSC activity regulation (11). In cases of breast cancer with BRCA1 mutations, activated RANKL/RANK signaling has been reported to increase CSC expansion (159). Further, WNT4, the most typical WNT ligand, can promote the action of progesterone. The WNT/β-catenin signaling pathway is a classical pathway for regulating CSCs. Progesterone stimulates PR^−^ cells through paracrine signals to produce WNT4 receptors, such as the coreceptor LRP5/6 and the cognate receptor frizzled protein (FZD) (34, 35, 160). The WNT4 ligand binds to the cysteine-rich domain of FZD and simultaneously binds to coreceptor LRP5/6 to activate the WNT/β-catenin pathway (32, 161). β-catenin is then released from the degradation complex, entering the nucleus and causing the expression of target genes (162). In T47D breast cancer cells, progesterone activates and upregulates the Notch pathway, which participates in CSC self-renewal (163). A recent study suggested that fallopian tube epithelial cells (FTECs) are the origin of epithelial ovarian cancer and that they can increase the expression of stemness genes (WNT and Notch) post treatment with estradiol or progesterone (164). Interestingly, in a study involving a mouse model, the activation of the Notch pathway in ovaries inhibited progesterone and estrogen secretion (165). This result was also confirmed by another study, which reported that the inhibition of the Notch signal stimulated progesterone secretion (166). This could be because there exists a protective negative feedback mechanism in normal tissues to prevent the overactivation of the Notch pathway. Progesterone-mediated CXC chemokine receptor type 4 (CXCR4) signaling is another paracrine pathway that regulates CSC expansion (Figure 2). The CXCR4 receptor and its CXC chemokine ligand 12 (CXCL12) are key mediators of progesterone-induced normal breast stem/progenitor cell functions (167). It has been confirmed that the CXCR4/CXCL12 pathway can maintain and promote prostate CSCs and enhance radiotherapy resistance (168).

## Progesterone Promotes the Generation of CSCs by Downregulating miRNA

miRNA is a type of non-coding small RNA that affects cancer stemness by regulating gene expression at the transcriptional level (169). Estrogen upregulates the expression of miR-29, which inhibits self-renewal and promotes differentiation; by contrast, progesterone downregulates the expression of miR-29 to enhance CSC characteristics (17) (Figure 2). This downregulation of miR-29 expression by progesterone leads to an increase in the protein level of the transcription factor KLF4, which is necessary for maintaining the pluripotency of CSCs and embryonic stem cells (18). Further, the downregulation of the expression of miR-29 enhances the expansion of CK5^+^ and CD44^+^ cancer cells, resulting in increased stem-like properties *in vitro* and *in vivo* (170). Progesterone has also been reported to downregulate the expression of miR-141, a member of the miR-200 family of tumor suppressors, leading to an increase in the number of stem-like breast cancer cells (CK5^+^ and CD44^+^ cells) (Figure 2). Further, the downregulation of miR-141 expression can upregulate the activator of transcription 5A (STAT5A), which is important for mammary stem cell expansion (144).

## Roles of Prolactin and GH IN Progesterone-Induced CSCs

The regulation of CSCs by progesterone is affected by prolactin and GH (Figure 2). The B-cell lymphoma-6 (BCL6) gene is an oncogene and a transcriptional repressor that plays a key role in maintaining leukemia stem cells. The upregulation of BCL6 induced by progesterone can promote the generation of the CK5^+^ stem cell population (47, 171). However, prolactin can inhibit the expression of BCL6 induced by progesterone (47). Further, progesterone stimulates the secretion of GH in human breast epithelial cells to increase the proliferation of GH receptor^+^ stem/progenitor breast cells (145). In addition, GH can reportedly promote BCSC properties and enhance cancer migration and invasion (172).

## Blockade of Cancer Stemness by Targeting the Progesterone Axis

Considering the mechanisms by which progesterone promotes CSCs, targeting PRs or downstream effectors of paracrine pathways is a reasonable treatment strategy. A study reported that mifepristone, an anti-progesterone drug that competes with progesterone for PRs, can be used for treating chemotherapy-resistant TNBC (173). Moreover, mifepristone reduces the number of CSCs in cases of TNBC (174). Another study found that mifepristone inhibits the proliferation, migration, and invasion of endometrial cancer cells by blocking the PI3K/AKT pathways (175). Further, a recent genome-wide RNAi study demonstrated that mifepristone is one of the best drugs for inhibiting CSCs (176). In the same study, by integrating RNAi screening results and functional mapping of CSC processes, the authors uncovered some potential therapeutic targets that could regulate the fate of BCSCs. They used a panel of 15 drugs to test these targets and found that mifepristone, salinomycin, and JQ1 showed the best anti-CSC activity. Onapristone is another selective PR antagonist that prevents PR-mediated transcription. It inhibits the nuclear translocation of phosphorylated PR (S294) (177). Herein, we earlier discussed that PR-A Ser294 phosphorylation is necessary for CSC development. Therefore, onapristone can disrupt the activities of CSCs by inhibiting the phosphorylation of PR-A Ser294. This conclusion was verified by a study that reported that the combination of onapristone and the FOXO1 inhibitor AS1842856 prevented the formation of tumorspheres in breast cancer cells (143). Besides, as mentioned earlier, activated RANKL/RANK signaling can affect CSC expansion. Therefore, denosumab, an anti-RANKL monoclonal antibody, can inhibit the progression of lung cancer by blocking the RANKL/RANK signaling pathway (178). It has also been demonstrated that the inhibition of RANK signaling markedly reduces the CSC pool and reduces tumor recurrence in the case of breast cancer (179). Hence, treatment with denosumab may inhibit progesterone-mediated CSC activity. In breast cancer with BRCA1 mutations, metformin inhibits RANKL and sensitizes CSCs to denosumab (180). Therefore, the combination of metformin and denosumab appears to be an effective treatment strategy.

To summarize, the potential of targeting the progesterone-induced axis to inhibit CSCs has been proved in preclinical models; future clinical studies should validate pertinent preclinical data. Progesterone is produced by the human body and cannot be targeted by drugs. The present anti-progesterone drugs function by binding to PRs. However, such progesterone antagonists cannot completely block progesterone-mediated CSC activity. Thus, using a combination of drugs that target PRs and the downstream mediators of PR signaling appears to be a better strategy.

## Conclusions and Perspectives

Targeting CSCs requires a better understanding of pertinent regulatory mechanisms. Both estrogen and progesterone signals have been shown to regulate CSCs, but many mechanisms remain to be comprehensively understood. Although conventional estrogen antagonists, progesterone modulators, and blocking agents of downstream pathways have been found to inhibit CSC activity in preclinical models, several issues still need to be resolved. First, many regulatory pathways are common between CSCs and normal stem cells, and thus, it is difficult to control side effects when targeting these pathways in a clinical setting. In addition, targeting one of the pathways in the regulatory networks of CSCs may activate other pathways, leading to the persistent generation of more CSCs. To comprehensively elucidate the downstream regulatory mechanisms of the estrogen and progesterone axis, sequencing a single tumor cell with stemness properties for the complete CSC gene map may be a better approach to discover a relatively complete regulatory network (181).

## Author Contributions

The manuscript was written and edited by BC, PY, YC, XY, and W-HY. TL and J-HC reviewed and edited the manuscript. XY and W-HY supervised the entire work. All authors agree to be responsible for the publication.

## Conflict of Interest

The authors declare that the research was conducted in the absence of any commercial or financial relationships that could be construed as a potential conflict of interest.
